# Changes of dental anxiety, aesthetic perception and oral health-related quality of life related to influencing factors of patients’ demographics after anterior implant treatment: a prospective study

**DOI:** 10.1186/s40729-023-00486-y

**Published:** 2023-08-02

**Authors:** Xin Xie, Zhengchuan Zhang, Jing Zhou, Feilong Deng

**Affiliations:** 1 Department of Oral Implantology, Hospital of Stomatology, Guanghua School of Stomatology, Sun Yat-Sen University, No. 56 of LingYuanXiLu, Guangzhou, 510055 Guangdong China; 2 Guangdong Provincial Key Laboratory of Stomatology, Guangzhou, China; 3 Department of Prosthodontics, Hospital of Stomatology, Guanghua School of Stomatology, Sun Yat-Sen University, No. 56 of LingYuanXiLu, Guangzhou, 510055 Guangdong China; 4 Department of Stomatology, The First Affiliated Hospital/The First Clinical Medicine School of Guangdong Pharmaceutical University, Guangzhou, China

**Keywords:** Dental anxiety, Aesthetic perception, Oral health-related quality of life, Anterior implant treatment

## Abstract

**Background:**

Accumulating evidence has revealed the effects of anterior implant procedures on dental anxiety (DA), aesthetic perception and oral health-related quality of life (OHRQoL). However, few reported the changes and influencing factors of the above outcomes before and after anterior implant treatment. This study was to evaluate the changes of DA, aesthetic perception and OHRQoL related to influencing factors of patients' demographics after anterior implant treatment.

**Methods:**

Thirty-nine patients satisfying the inclusion criteria were prospectively recruited before surgery. The subjects completed the Modified Dental Anxiety Scale (MDAS), the Orofacial Esthetic Scale (OSE) and the Oral Health Impact Profile-14 (OHIP-14), before implant surgery and after definitive prosthesis placement. Mann–Whitney *U* test and Kruskal–Wallis test by Bonferroni correction were applied for the data analysis and the influencing factors evaluation (*p* < 0.05).

**Results:**

Overall, 39 patients (mean age of 44.9 ± 12.0) completed the three scales. After anterior implant treatment, MDAS was not significantly changed (*p* > 0.05). The overall OSE (*p* < 0.001) and OHIP-14 (*p* < 0.05) were significantly improved. Females showed more improvement of overall OHIP score than males after anterior implant treatment (*p* < 0.05).

**Conclusions:**

Anterior implant procedures did not change the level of patient’s DA, while aesthetic perception and OHRQoL were enhanced. Only gender difference of overall OHIP change was found in our study. Thus, more related influencing factors with larger sample and long-term effective follow-up are needed.

*Trial registration*: ClinicalTrials.gov, NCT05424458. Registered 13 June 2022—Retrospectively registered, https://clinicaltrials.gov/ct2/show/NCT05424458.

## Introduction

The critically demanding task for aesthetic restorations of anterior missing teeth poses a challenge to a successful implant treatment [1]. Accumulating evidence has revealed the positive effects of anterior implant procedures on dental anxiety (DA), aesthetic perception and oral health-related quality of life (OHRQoL) [2–5]. However, the changes of DA, aesthetic perception and OHRQoL under different demographics were few reported.

Moderate-to-high level of perioperative DA has been reported in many studies, which may have a negative influence on the physical and psychological experience of implant treatment satisfaction [3, 6]. Besides, the fear of dental surgery may get patients to refuse implant solutions and turn to accept conventional dentures, which will lead to the compromised aesthetic and functional rehabilitation of the anterior teeth [7]. These results may also lead to an endless loop of unsatisfied experience and continuing high DA level. However, whether the anterior implant treatment could change the DA level of patients is unclear.

Anterior implant treatment may yield preferable aesthetic outcomes [8]. Aesthetic perception, one kind of self-perception, is suitable to evaluate the result of patient-centred treatment [9]. Implant treatment will change the orofacial aesthetics characteristics, which may cause the changes of self-perception outcomes [5]. Besides, OHRQoL must be taken seriously enough to evaluate clinical interventions [10]. Changes of OHRQoL by the anterior implant treatment have been showed more radical than the posterior implant treatment [11, 12]. It was reported that a high aesthetics and function satisfaction after both definitive prosthesis placement and 10-year follow-up [2, 13]. Thus, the earliest and most significant improvement of OHRQoL might be the time of the definitive restoration placement.

However, patient’s perception may be influenced by their individuality. Patients may not show the same self-perception and psychosocial impacts because of different demographics, such as age, gender, educational status, tooth loss number, smoking habit, simultaneously bone augmentation, loading timing and prosthesis type [14, 15]. Previous studies have showed higher occurrence of tooth loss by periodontitis and subsequent implant failure in patients with smoking habit, which may cause a contradictory psychological need for the balance of the anterior implant treatment and the addiction to smoking [16, 17]. Anterior implants involve the comprehensive solutions of surgery and prosthetics, such as simultaneously bone augmentation, loading timing and prosthesis type [18, 19]. However, whether these influencing factors of patients' demographics could change DA, aesthetic perception and OHRQoL after anterior implant treatment is unclear.

The purpose of this prospective study was to evaluate the changes of DA, aesthetic perception and OHRQoL related to influencing factors of patients' age, gender, educational status, tooth loss number, smoking habit, simultaneously bone augmentation, loading timing and prosthesis type after anterior implant treatment.

## Materials and methods

### Study design

The present study was designed as a prospective study and reported according to STROBE guideline. The study protocol was approved by the Medical Ethics Committee of Hospital of Stomatology, Sun Yat-sen University (KQEC-2021-46-01) and conducted in full accordance with the Helsinki declaration of 1975 and revised in 2013.

### Patient selection

The subjects (*n* = 39) included in this prospective study were recruited from those patients with anterior missing teeth at the Department of Oral Implantology, Guanghua School of Stomatology, Sun Yat-sen University.

Patients satisfying the following inclusion criteria were recruited: (1) age ≥ 18 years old; (2) partially anterior edentulous jaws; (3) patients will be given an anterior implant surgery and implant-supported fixed rehabilitation; (4) patients could express themselves and communicate normally; (5) willing to participate in and accept investigation. Exclusion criteria were (1) use of anti-anxiety and painkillers within 1 year; (2) mental and psychological diseases with poor emotional self-control; (3) a history of previous implant loss; (4) ongoing active infections by endodontic or periodontal problems of all the remaining teeth; (5) combined complex surgery, such as large-block autogenous bone grafting; (6) severe systemic diseases influencing implant survival (uncontrolled diabetes mellitus, previous chemotherapy, previous irradiation of the head and neck region, immunosuppression, etc.).

### Data collection

Before implant surgery, all participants signed an informed consent form and were given sufficient time in the waiting room to answer the following three scales (Table 1). The modified dental anxiety scale (MDAS) included five questions with a 5-category scale, ranging from ‘not’ to ‘extremely' [20]. The Orofacial Esthetic Scale (OES) was a scale that was designed by 8 items to evaluate the self-perception of aesthetic implant treatment (ranged from 0 to 10 scores, 0 is ‘Very dissatisfied’ and 10 is ‘Very satisfied’) [21]. The Oral Health Impact Profile (OHIP) was to measure OHRQoL, comprising 14 statements with 5 scores (1 = Not, 2 = Seldom, 3 = Sometimes, 4 = Often, 5 = Very often; total scores: 14–70) [10]. Influencing factors of patients' demographics including age, gender, educational status, tooth loss number and smoking habit were obtained from the medical records.

**Table 1:** The MDAS, OSE and OHIP questionnaires

MDAS questionnaires
Q1 How anxious would you feel if you prepared to see a dentist at home?
Q2 How anxious would you feel if you were waiting for treatment in the waiting room?
Q3 Have you felt anxious when the dentist drilled your teeth with a dental drill?
Q4 Have you felt anxious when you were about to have your teeth treated?
Q5 Have you felt anxious when you saw the anesthetic needle in your mouth?
OSE questionnaires
Q1 Frontal appearance of face
Q2 Appearance of facial profile
Q3 Mouth appearance (smile, lips and visible teeth)
Q4 Appearance of teeth rows
Q5 Teeth shape
Q6 Teeth colour
Q7 Gum appearance
Q8 Overall, how do you feel about the appearance of your face, mouth and teeth?
OHIP questionnaires
Q1 Have you felt pronunciation problem because of your teeth?
Q2 Have you felt less tasty of food because of your teeth?
Q3 Have you felt painful areas in your mouth?
Q4 Have you felt that your appearance has been affected by missing teeth?
Q5 Have you been uncomfortable in public because of your teeth?
Q6 Have you been nervous because of your teeth?
Q7 Have you been unsatisfied with food because of your teeth?
Q8 Have you had to stop eating because of your teeth?
Q9 Have you been difficult to relax because of your teeth?
Q10 Have you been embarrassed because of teeth?
Q11 Have you been temperish because of teeth?
Q12 Have you had difficulties doing your usual job because of teeth?
Q13 Have you felt that life was less satisfying because of teeth?
Q14 Have you felt that you could do nothing because of teeth?

On MDAS questionnaire numbers correspond to the dimensions (1 = Not, 2 = Slightly, 3 = Fairly, 4 = Very, 5 = Extremely. Overall MDAS score: Q1–Q5 summary score)

On OSE questionnaire numbers correspond to the dimensions (ranged from 0 to 10, 0 is ‘Very dissatisfied’ and 10 is ‘Very satisfied’)

On OHIP questionnaire numbers correspond to the dimensions (1 = Not, 2 = Seldom, 3 = Sometimes, 4 = Often, 5 = Very often)

In translation to Chinese for patients use

### Clinical procedures

Patients received routine examinations before surgery. The surgical procedures were performed by experienced experts. Immediate loading protocol was delivered if the insertion torque was over 35 N·cm; otherwise, removable restorations with submerged implants were applied. After a healing period of 3–6 months, a definitive screw-retained porcelain-fused-to-metal (PFM) or a CAD/CAM zirconia restoration were performed.

In the first month after definitive prosthesis placement, patients were recalled to complete the MDAS, OES and OHIP questionnaires for the second time. Changes of overall MDAS, OSE and OHIP scores were defined as the score after definitive prosthesis placement minus that before the treatment. Negative score changes indicated score decrease of the second questionnaire compared to the first one. Positive score changes indicated score increase.

### Statistical analysis

The surgeon, prosthetist and nurse of all the enrolled patients were the same and consistent. Data were collected and evaluated from the scales by two independent researchers. Data were calculated by descriptive statistics (mean, standard deviation) and were analysed using the SPSS 25.0 software package (SPSS Inc., USA). Mann–Whitney *U* test was used to determine the score change before and after anterior implant treatment. Mann–Whitney U test (gender, tooth loss number, smoking habit, simultaneously bone augmentation, loading timing and prosthesis type) and Kruskal–Wallis test by Bonferroni correction (age and educational status) were applied for the influencing factors evaluation based on the changes of overall MDAS, OSE and OHIP scores. The level of significance was set at *p* < 0.05.

## Results

### Patient’s demographics

**Fig. 1:**
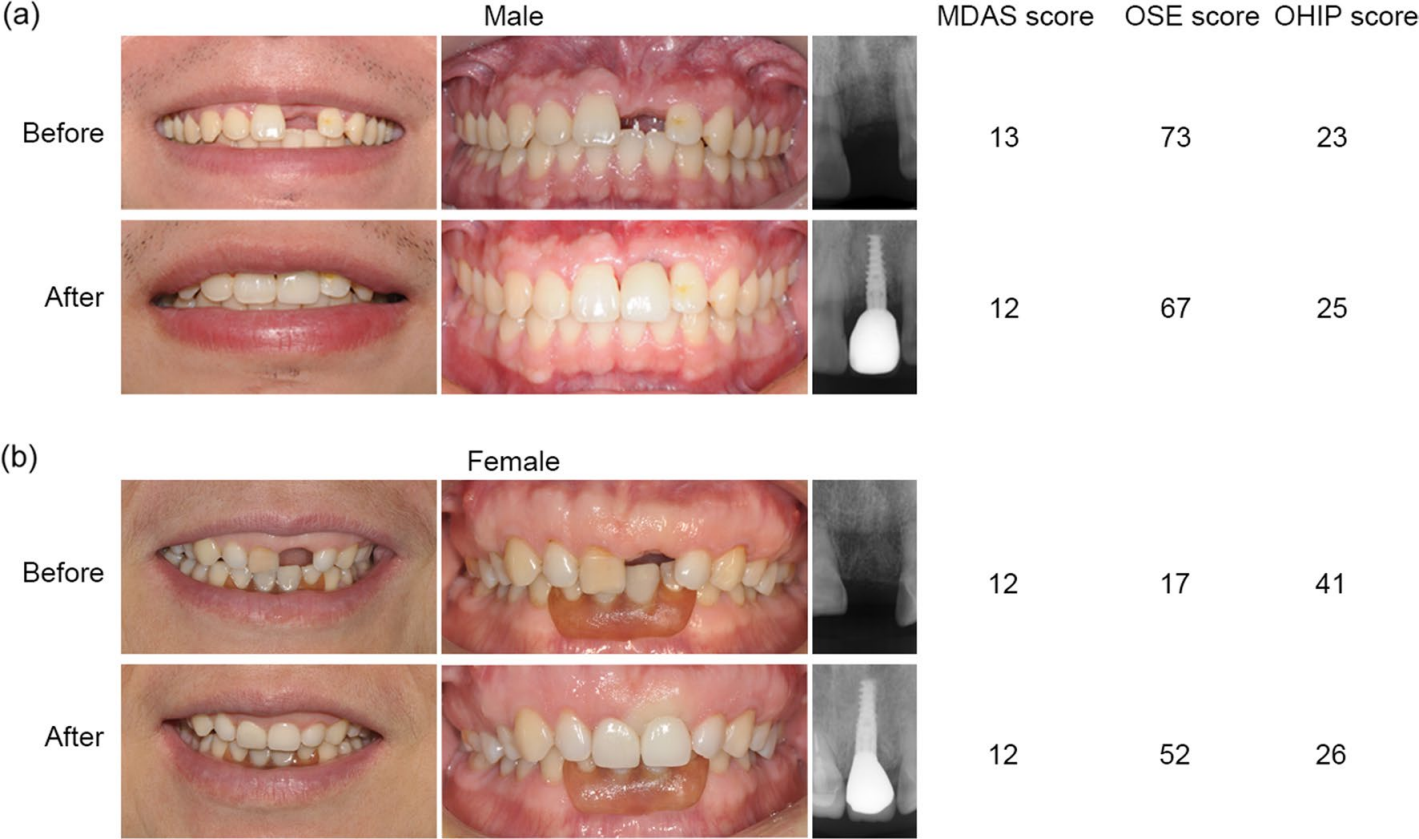
Representative photographs and scores with different genders. **a** The extraoral, intraoral, radiographic photographs and the scores of a male patient before and after implant treatment. **b** The extraoral, intraoral, radiographic photographs and the scores of a female patient before and after implant treatment

A total of 39 patients were enrolled and evaluated. The mean time intervals between two-time questionnaire were 9.8 ± 2.6 months. The mean age of the patients during the surgery was 44.9 ± 12.0 years ranged from 24 to 70 years. Over half of all the participants (56.4%) were middle-aged (40 < age ≤ 60). Males accounted for 56.4%, slightly higher than females (43.6%). From the aspect of educational status, 87.2% of patients had a college degree or above. The number of tooth loss was 2.0 ± 1.5 with 56.4% portion for single tooth loss. Only 17.9% of patients had smoking habit. Implant placement with simultaneously bone augmentation accounted for 56.4%. Most of the enrolled patients received delayed loading with zirconia definitive restorations (Table 2). The extraoral, intraoral, radiographic photographs and the scores with different genders before and after implant treatment are exhibited as Fig. 1.

**Table 2:** Changes of overall MDAS, OSE and OHIP score related to influencing factors of patients' demographics

Variable	No. patients (%)	Change of overall MDAS score		Change of overall OSE score		Change of overall OHIP score	
		Mean ± SD	*p*-value	Mean ± SD	*p*-value	Mean ± SD	*p*-value
Age	44.9 ± 12.0 [24–70]						
Age ≤ 40	13 (33.3%)	− 0.8 ± 3.2	0.359	27.2 ± 32.4	0.129	− 4.1 ± 8.7	0.189
40 < age ≤ 60	22 (56.4%)	− 1.1 ± 5.7		36.4 ± 29.2		− 6.6 ± 14.9	
Age > 60	4 (10.3%)	2.0 ± 2.7		63.0 ± 19.6		− 12.3 ± 7.4	
Gender							
Male	22 (56.4%)	− 0.7 ± 4.1	0.432	29.0 ± 30.7	0.076	− 2.5 ± 8.5	0.047*
Female	17 (43.6%)	− 0.6 ± 5.6		45.2 ± 28.8		− 11.3 ± 15.1	
Educational status							
None/primary/middle school	5 (12.8%)	− 1.4 ± 3.0	0.780	39.2 ± 34.2	0.126	− 7.0 ± 8.1	0.761
College/undergraduate	27 (69.2%)	− 0.6 ± 5.6		41.3 ± 31.5		− 6.8 ± 14.5	
MS/PhD	7 (18.0%)	− 0.4 ± 1.5		13.7 ± 11.1		− 4.1 ± 5.6	
Tooth loss number							
Single	22 (56.4%)	− 0.9 ± 5.2	0.898	36.2 ± 31.3	0.910	− 9.9 ± 14.1	0.063
Multiple	17 (43.6%)	− 0.4 ± 4.2		35.8 ± 30.6		− 1.8 ± 8.3	
Smoking habit							
Yes	7 (17.9%)	− 0.9 ± 3.5	0.386	44.6 ± 27.5	0.410	− 5.7 ± 7.3	0.840
No	32 (82.1%)	− 0.6 ± 5.0		34.2 ± 31.3		− 6.5 ± 13.4	
Simultaneously bone augmentation							
Yes	22 (56.4%)	− 0.3 ± 4.0	0.764	29.3 ± 30.0	0.076	− 3.0 ± 9.5	0.069
No	17 (43.6%)	− 1.1 ± 5.6		44.8 ± 29.9		− 10.6 ± 14.7	
Loading timing							
Immediate	4 (10.3%)	− 0.5 ± 8.2	0.981	35.5 ± 25.0	0.945	− 9.5 ± 12.9	0.404
Delayed	35 (89.7%)	− 0.7 ± 4.4		36.1 ± 31.4		− 6.0 ± 12.6	
Prosthesis type							
Zirconia	36 (92.3%)	− 0.8 ± 4.8	0.832	36.4 ± 30.6	0.792	− 6.2 ± 13.0	0.178
PFM	3 (7.7%)	0.7 ± 5.0		32.3 ± 36.6		− 8.0 ± 1.7	

*p < 0.05 compared to other variable items by Mann–Whitney *U* test (gender, tooth loss number, smoking habit, simultaneously bone augmentation, loading timing and prosthesis type) and Kruskal–Wallis test by Bonferroni correction (age and educational status)

### Dental anxiety

Dental anxiety levels were assessed by MDAS (Table 3). Before anterior implant treatment, the overall MDAS score was 11.9 ± 4.2 (slightly to fairly anxious). After definitive prosthesis placement, the overall MDAS score was 11.3 ± 4.8 (slightly to fairly anxious). Differences of overall DA levels between the two time points were not significant (*p* = 0.388). There were almost no obvious score changes of Q1–Q5 during the treatment. A relatively low level of anxiety was observed when the patients were waiting for treatment in the waiting room (not to slightly anxious) compared the other four situations from the 5-category scale.

**Table 3:** Distribution of scores by dimension and for the total MDAS (mean ± SD)

	Before implant treatment	After implant treatment	*p*-value
Q1	2.8 ± 1.3	2.4 ± 1.3	0.243
Q2	1.5 ± 0.9	1.5 ± 0.9	0.917
Q3	2.7 ± 1.1	2.8 ± 1.1	0.847
Q4	2.1 ± 1.1	1.9 ± 1.1	0.319
Q5	2.8 ± 1.1	2.7 ± 1.2	0.598
Overall MDAS score	11.9 ± 4.2	11.3 ± 4.8	0.388

*p < 0.05 compared to the scores before implant treatment by Mann–Whitney *U* test

### Aesthetic perception

The Orofacial Esthetic Scale (OES) was designed to evaluate the aesthetic perception changes (Table 4). All the subitem’s scores of OSE and the overall OSE score after implant treatment displayed significant increase than those before implant treatment (*p* < 0.001). Based on the change of the overall OSE score, doubled aesthetic perception level was achieved after the anterior implant treatment by the patient-reported OSE outcomes.

**Table 4:** Distribution of scores by dimension and for the total OSE (mean ± SD)

	Before implant treatment	After implant treatment	*p*-value
Q1	4.9 ± 3.6	9.2 ± 1.1	< 0.001*
Q2	5.1 ± 3.5	9.2 ± 1.1	< 0.001*
Q3	4.1 ± 3.6	8.7 ± 2.0	< 0.001*
Q4	4.1 ± 3.5	9.0 ± 1.3	< 0.001*
Q5	4.1 ± 3.7	8.9 ± 1.4	< 0.001*
Q6	4.4 ± 3.6	8.9 ± 1.4	< 0.001*
Q7	4.3 ± 3.6	8.8 ± 1.5	< 0.001*
Q8	4.9 ± 3.3	9.1 ± 1.2	< 0.001*
Overall OSE score	35.9 ± 27.0	71.9 ± 9.6	< 0.001*

*p < 0.05 compared to the scores before implant treatment by Mann–Whitney *U* test

### Oral health-related quality of life

The Oral Health Impact Profile (OHIP) survey score was to measure oral health-related quality of life (Table 5). In total, the overall OHIP scores of the two time points were seldom to sometimes level in the both two time points. Statistically significant improvements were found after definitive prosthesis placement in the following dimensions: pronunciation problem (*p* = 0.009), eating disorders (*p* = 0.048), social obstacles (*p* = 0.003), emotional control (*p* = 0.034), usual job (*p* = 0.016), life satisfaction (*p* = 0.006) and confidence to do things (*p* = 0.036), as well as in the overall OHIP score (*p* = 0.020).

**Table 5:** Distribution of scores by dimension and for the total OHIP (mean ± SD)

	Before implant treatment	After implant treatment	*p*-value
Q1	2.7 ± 1.2	2.0 ± 1.1	0.009*
Q2	2.1 ± 1.2	1.6 ± 1.0	0.141
Q3	2.2 ± 1.0	1.8 ± 1.0	0.183
Q4	3.6 ± 1.2	3.3 ± 1.4	0.490
Q5	3.3 ± 1.2	2.8 ± 1.4	0.127
Q6	2.9 ± 1.2	2.6 ± 1.2	0.176
Q7	2.6 ± 1.2	2.1 ± 1.0	0.101
Q8	2.4 ± 1.1	1.9 ± 0.9	0.048*
Q9	2.2 ± 1.1	1.9 ± 1.0	0.385
Q10	3.0 ± 1.0	2.3 ± 1.1	0.003*
Q11	1.8 ± 1.0	1.4 ± 0.6	0.034*
Q12	1.8 ± 1.0	1.3 ± 0.6	0.016*
Q13	2.5 ± 1.2	1.8 ± 0.9	0.006*
Q14	1.6 ± 0.8	1.3 ± 0.6	0.036*
Overall OHIP score	34.6 ± 11.4	28.2 ± 9.7	0.020*

*p < 0.05 compared to the scores before implant treatment by Mann–Whitney *U* test

### Influencing factors of patients' demographics

Table 2 shows the influencing factors of patients' demographics including age, gender, educational status, tooth loss number, smoking habit, simultaneously bone augmentation, loading timing and prosthesis type. Age and educational status were set as three categories. There were no significant differences of MDAS, OSE and OHIP score changes among age, educational status, tooth loss number, smoking habit, simultaneously bone augmentation, loading timing and prosthesis type. Females showed more improvement of overall OHIP score change than males (*p* = 0.047), while no significant differences were observed between males and females in MDAS (*p* = 0.432) and OSE score (*p* = 0.076), respectively.

## Discussion

This prospective study focused on the changes of DA, aesthetic perception and OHRQoL related to influencing factors of patients' age, gender, educational status, tooth loss number, smoking habit, simultaneous bone augmentation, loading timing and prosthesis type after anterior implant treatment. After anterior implant treatment, MDAS were not significantly changed (*p* > 0.05), while the overall OSE (*p* < 0.001) and OHIP-14 (*p* < 0.05) were significantly improved. Females showed more improvement of overall OHIP score than males (*p* < 0.05). Meanwhile, age, educational status, tooth loss number, smoking habit, simultaneously bone augmentation, loading timing and prosthesis type did not exhibit significant changes in MDAS, OSE and OHIP.

Slightly to fairly anxious DA levels in oral implant patients were reported both before anterior implant treatment and after definitive prosthesis placement in our study. This study reported DA prevalence of 69.2% and 66.7% for the patients before the treatment and after the treatment, respectively, in accordance with previous studies with high prevalence in oral surgery patients [3, 6]. High prevalence of DA may be not beneficial to comprehensive implant treatment, especially in the aesthetic or anterior zone. It was reported that the fear of surgery was the most common reason to avoid implants [7]. With the advent of the information age and big data era, multimedia information about the details of implant surgery was easily obtained and impressed for the patients, which led to high preoperative DA level without intervention of dental professionals [22]. After definitive prosthesis placement, DA changes were not found in the present study. High DA level was associated with enhanced pain perception; thus, DA level did not decrease after the surgery procedures with more or less surgery related pain [3]. Therefore, it is necessary to release anxiety in patients, especially during the first dental implant surgery. Preoperative psychological intervention and perioperative classical era music may help patients to decrease dental anxiety and pain perception [23, 24].

Greatly improved after-treatment rating of aesthetic perception based on the OES was achieved in the patient with implant-supported fixed anterior restorations in the present study. High overall satisfaction of face appearance, mouth appearance and teeth appearance was easily obtained with anterior implant treatment in comparison with conventional dentures [5]. Enhanced confidence from the improved oral function further promoted the positive aesthetic perception during the follow-up. Therefore, doubled scores of the aesthetic perception after definitive prosthesis placement confirmed the positive effect of anterior implant treatment on the patient-reported overall outcomes. A definitive screw-retained porcelain-fused-to-metal (PFM) or a CAD/CAM zirconia restoration were performed with lingual screw access hole as invisible as possible, which satisfied patients’ aesthetic needs [25].

In the current study, OHIP-14 results revealed a statistically significant improvement of OHRQoL in many aspects. The second questionnaires were made in the first month after definitive prosthesis placement with better perception of the treatment, without the interference of the most unfavourable OHIP scores of healing period [4, 26–28]. This study did not group the different implant timings, because the effects of different surgical solutions shared similar clinical procedures from the perspectives of patients in the time point after the definitive restoration [29, 30]. The time intervals of the present study were not fixed, which were different for varied healing periods of patients. The aim of this design was to emphasize the importance of the implant restorations in the short-term observation. Based on the previous study, a very high and stable long-term overall satisfaction was achieved regarding physical and psychological experience for 10 years after implant placement [2, 31].

To our surprise, we found that only gender differences of overall OHIP change were found after definitive prosthesis placement in our study. However, age, educational status, tooth loss number, smoking habit, simultaneously bone augmentation, loading timing and prosthesis type did not exhibit significant changes in MDAS, OSE and OHIP, which were also reported in other studies [3, 15]. Women were reported to be more attentive to oral health issues with the stronger desire to choose aesthetic treatment than men [32, 33]. Therefore, women may benefit from anterior implant treatment with favourable aesthetics and then report a better OHIP score.

The present study has limitations that should be taken into consideration when interpreting the findings. The sample size of the present study was calculated based on the level of DA, aesthetic perception and OHRQoL of implant patients from the previous studies, without consideration of related influencing factors [3, 5]. Thus, population distributions of age, gender, educational status, tooth loss number, smoking habit, simultaneously bone augmentation, loading timing and prosthesis type were not average in their subitems. Difference between groups may be influenced by minorities, such as age over 60 (10.3%), none/primary/middle school educational status (12.8%), immediate loading (10.3%) and PFM restoration (7.7%). Hence, a randomized controlled study design with a specific patients' demographic will be necessary to discover the response bias and substantiate the present study.

## Conclusion

Within the limitations of the study, anterior implant procedures did not change the level of patient’s DA, while aesthetic perception and OHRQoL were improved. Only gender differences of overall OHIP change were found in our study. Besides, more related influencing factors with larger sample and long-term effective follow-up are needed.

## Data Availability

All data generated or analysed during this study are included in this published article.

## Abbreviations

DA: Dental anxiety
OHRQoL: Oral health-related quality of life
MDAS: Modified Dental Anxiety Scale
OSE: Orofacial Esthetic Scale
OHIP-14: Oral Health Impact Profile-14
PFM: Porcelain-fused-to-metal

## References

[1] Kan JYK, Rungcharassaeng K, Deflorian M, Weinstein T, Wang HL, Testori T (2018). Immediate implant placement and provisionalization of maxillary anterior single implants. Periodontol 2000.

[2] Wang Y, Baumer D, Ozga AK, Korner G, Baumer A (2021). Patient satisfaction and oral health-related quality of life 10 years after implant placement. BMC Oral Health.

[3] Zhang X, Wang B, Qiao SC, Gu YX, Shi JY, Lai HC (2019). A study on the prevalence of dental anxiety, pain perception, and their interrelationship in Chinese patients with oral implant surgery. Clin Implant Dent Relat Res.

[4] Yoshida T, Masaki C, Komai H, Misumi S, Mukaibo T, Kondo Y (2016). Changes in oral health-related quality of life during implant treatment in partially edentulous patients: a prospective study. J Prosthodont Res.

[5] Persic S, Celebic A (2015). Influence of different prosthodontic rehabilitation options on oral health-related quality of life, orofacial esthetics and chewing function based on patient-reported outcomes. Qual Life Res.

[6] Bovaira M, Herrero Babiloni A, Jovani M, Penarrocha-Diago M, Gonzalez-Lemonnier S, Penarrocha-Oltra D (2017). Preoperative anxiety and its influence on patient and surgeon satisfaction in patients receiving dental implant surgeries performed under intravenous conscious sedation. Int J Oral Maxillofac Implants.

[7] Elfadil S, Johnston B, Normand C, Allen F, O'Connell B (2021). An investigation of the characteristics of edentulous patients who choose or refuse implant treatment. Int J Prosthodont.

[8] Sanchez-Perez A, Nicolas-Silvente AI, Sanchez-Matas C, Molina-Garcia S, Navarro-Cuellar C, Romanos GE (2021). Primary stability and PES/WES evaluation for immediate implants in the aesthetic zone: a pilot clinical double-blind randomized study. Sci Rep.

[9] Bersezio C, Martin J, Herrera A, Loguercio A, Fernandez E (2018). The effects of at-home whitening on patients' oral health, psychology, and aesthetic perception. BMC Oral Health.

[10] Nielsen HB, Schou S, Bruun NH, Starch-Jensen T (2022). Professional and patient-reported outcomes of two surgical approaches for implant-supported single-crown restoration: 1-year results of a randomized controlled clinical trial. Clin Oral Implants Res.

[11] Hara M, Matsumoto T, Yokoyama S, Higuchi D, Baba K (2017). Location of implant-retained fixed dentures affects oral health-related quality of life. Clin Implant Dent Relat Res.

[12] Nickenig HJ, Wichmann M, Terheyden H, Kreppel M (2016). Oral health-related quality of life and implant therapy: a prospective multicenter study of preoperative, intermediate, and posttreatment assessment. J Craniomaxillofac Surg.

[13] Machuca C, Vettore MV, Robinson PG (2020). How peoples' ratings of dental implant treatment change over time?. Qual Life Res.

[14] AlZarea BK (2017). Oral health related quality-of-life outcomes of partially edentulous patients treated with implant-supported single crowns or fixed partial dentures. J Clin Exp Dent.

[15] Yu SJ, Chen P, Zhu GX (2013). Relationship between implantation of missing anterior teeth and oral health-related quality of life. Qual Life Res.

[16] Raabe C, Monje A, Abou-Ayash S, Buser D, von Arx T, Chappuis V (2021). Long-term effectiveness of 6 mm micro-rough implants in various indications: a 4.6- to 18.2-year retrospective study. Clin Oral Implants Res.

[17] Levin L, Schwartz-Arad D (2005). The effect of cigarette smoking on dental implants and related surgery. Implant Dent.

[18] Leonard JF, Taxel P, Kuo CL, Da Cunha Godoy L, Freilich M. Dental implant and bone augmentation treatment in bone-compromised patients: oral health-related quality of life outcomes. J Prosthet Dent. 2023. 10.1016/j.prosdent.2023.01.011 PMC10435665 36804393

[19] Rutkowski R, Smeets R, Neuhoffer L, Stolzer C, Strick K, Gosau M (2022). Success and patient satisfaction of immediately loaded zirconia implants with fixed restorations one year after loading. BMC Oral Health.

[20] Newton JT, Edwards JC (2005). Psychometric properties of the modified dental anxiety scale: an independent replication. Community Dent Health.

[21] Wetselaar P, Koutris M, Visscher CM, Larsson P, John MT, Lobbezoo F (2015). Psychometric properties of the Dutch version of the Orofacial Esthetic Scale (OES-NL) in dental patients with and without self-reported tooth wear. J Oral Rehabil.

[22] Kazancioglu HO, Dahhan AS, Acar AH (2017). How could multimedia information about dental implant surgery effects patients' anxiety level?. Med Oral Patol Oral Cir Bucal.

[23] Esteban Pellicer LA, Conde Villar AJ, Martinez Rubio JL, Casanas E, Estevez LR (2023). Can music decrease anxiety and pain during dental implant surgery? A randomized clinical trial. J Oral Maxillofac Surg.

[24] Lin X, Li S, Yang S, Zheng X (2022). Preoperative online intervention to dental anxiety in patients with transcrestal sinus floor elevation. Quintessence Int.

[25] Wen C, Jiang R, Zhang Z, Lei B, Zhong Y, Zhou H (2022). Esthetic evaluation and acceptability of different hole designs on implant crowns from the perspective of patients and dentists in China. Patient Prefer Adherence.

[26] Eitner S, Wichmann M, Schlegel KA, Kollmannsberger JE, Nickenig HJ (2012). Oral health-related quality of life and implant therapy: an evaluation of preoperative, intermediate, and post-treatment assessments of patients and physicians. J Craniomaxillofac Surg.

[27] Winter A, Erdelt K, Giannakopoulos NN, Schmitter M, Edelhoff D, Liebermann A (2021). Impact of different types of dental prostheses on oral-health-related quality of life: a prospective bicenter study of definitive and interim restorations. Int J Prosthodont.

[28] Farzadmoghadam M, Mohammadi TM, Goudarzi R, Mohammadi M, Hasheminejad N (2020). Is there a relationship between general and oral health-related quality of life in partially edentulous patients before and after implant treatment? A quasi-experimental study. Clin Oral Implants Res.

[29] Kunavisarut C, Santivitoonvong A, Chaikantha S, Pornprasertsuk-Damrongsri S, Joda T (2021). Patient-reported outcome measures comparing static computer-aided implant surgery and conventional implant surgery for single-tooth replacement: a randomized controlled trial. Clin Oral Implants Res.

[30] Raes F, Cosyn J, De Bruyn H (2013). Clinical, aesthetic, and patient-related outcome of immediately loaded single implants in the anterior maxilla: a prospective study in extraction sockets, healed ridges, and grafted sites. Clin Implant Dent Relat Res.

[31] Heydecke G, Mirzakhanian C, Behneke A, Behneke N, Fugl A, Zechner W (2019). A prospective multicenter evaluation of immediately functionalized tapered conical connection implants for single restorations in maxillary anterior and premolar sites: 3-year results. Clin Oral Investig.

[32] Margaryan EG, Paramonov YO (2017). Gender-related preferences in the choice of methods for aesthetic and functional rehabilitation in dentistry. Stomatologiia (Mosk).

[33] Baumgarten A, Bastos JL, Toassi RFC, Hilgert JB, Hugo FN, Celeste RK (2018). Discrimination, gender and self-reported aesthetic problems among Brazilian Adults. Community Dent Oral Epidemiol.

